# Nucleic Acids and Their Analogues for Biomedical Applications

**DOI:** 10.3390/bios12020093

**Published:** 2022-02-04

**Authors:** Fei Wang, Pan Li, Hoi Ching Chu, Pik Kwan Lo

**Affiliations:** 1 Department of Chemistry, City University of Hong Kong, Hong Kong SAR 999077, China; fwang55-c@my.cityu.edu.hk (F.W.); panli8-c@my.cityu.edu.hk (P.L.); hoicchu7-c@my.cityu.edu.hk (H.C.C.); 2 Key Laboratory of Biochip Technology, Biotech and Health Care, Shenzhen Research Institute of City University of Hong Kong, Shenzhen 518057, China

**Keywords:** nucleic acid, nucleic acid analogues, nanomaterials, DNA nanotechnology, biomedical applications, gene regulation, biosensing, drug delivery, therapy

## Abstract

Nucleic acids are emerging as powerful and functional biomaterials due to their molecular recognition ability, programmability, and ease of synthesis and chemical modification. Various types of nucleic acids have been used as gene regulation tools or therapeutic agents for the treatment of human diseases with genetic disorders. Nucleic acids can also be used to develop sensing platforms for detecting ions, small molecules, proteins, and cells. Their performance can be improved through integration with other organic or inorganic nanomaterials. To further enhance their biological properties, various chemically modified nucleic acid analogues can be generated by modifying their phosphodiester backbone, sugar moiety, nucleobase, or combined sites. Alternatively, using nucleic acids as building blocks for self-assembly of highly ordered nanostructures would enhance their biological stability and cellular uptake efficiency. In this review, we will focus on the development and biomedical applications of structural and functional natural nucleic acids, as well as the chemically modified nucleic acid analogues over the past ten years. The recent progress in the development of functional nanomaterials based on self-assembled DNA-based platforms for gene regulation, biosensing, drug delivery, and therapy will also be presented. We will then summarize with a discussion on the advanced development of nucleic acid research, highlight some of the challenges faced and propose suggestions for further improvement.

## 1. Introduction

Nucleic acids, including deoxyribonucleic acid (DNA) and ribonucleic acid (RNA), are natural biomacromolecules that exist universally in living organisms [1]. They play fundamental roles in many biological processes. In brief, DNA is the hereditary material that stores, encodes, and transfers genetic information to other components of cells, such as RNA and protein molecules. RNA serves as an intermediate messenger for gene expression by linking the DNA with protein synthesis machinery [2,3]. In addition, nucleic acids also exhibit other biological functions, such as catalysis of certain biochemical reactions and regulation of certain activities in cells [4]. In all cases, the fundamental basis of these biological functions is the ability of DNA/RNA to adopt complex three-dimensional (3D) structures and interconvert between different functional states [5]. Determining the structure flexibility of nucleic acids is a crucial factor for a detailed understanding of their specific functions.

Scientists have made tremendous strides in understanding the molecular structure and biological functions of nucleic acids. Consequently, nucleic acids have potential utilities in various biomedical applications, including gene regulation, sensing and detection, bioimaging, drug delivery, and disease therapy. There are currently several nucleic-acid-based drugs available in the market that have been approved by the U.S. Food and Drug Administration (FDA). In addition, many RNA interference (RNAi)- and antisense oligonucleotide (ASO)-based drugs are undergoing clinical trials, and arepromising for treatment of various disease [6].

However, the potential clinical translations of nucleic-acid-based drugs are still restricted due to the limitations of natural nucleic acids (DNA/RNA) such as stability, pharmacokinetics, distribution, and immunity. Natural nucleic acids are susceptible to nucleases and are rapidly degraded upon exposure in physiological conditions [7]. For example, a study showed that short single-stranded DNA (ssDNA) had a half-life of about 30 min in the presence of RQ1 DNase, while the corresponding RNA sample had a half-life of less than 10 s in the presence of RNase A [8]. Moreover, as a polyanion biomolecule, natural nucleic acids usually have limited cellular uptake and accumulation because they cannot efficiently penetrate cells by themselves due to their size and negative charge [9,10]. For in vivo studies, natural nucleic acids also have a very short half-life when circulating in blood. This is highly attributed to rapid intravascular degradation and renal clearance due to the dominant uptake by kidney and/or organs including liver, spleen, lymph nodes, adipocytes, and bone marrow after intravenous or subcutaneous injection [7]. Furthermore, natural nucleic acids may trigger an immune response and lead to severe autoimmune pathology. For example, small interfering RNAs (siRNAs) are potent activators of the mammalian innate immune system. Synthetic siRNA duplexes can trigger high levels of inflammatory cytokines and type I interferons after systemic administration [11]. These in vitro and in vivo properties result in lower therapeutic efficacy, potential off-target effects, and biosafety issues of nucleic-acid-based drugs, thus limiting their clinical translation.

Accordingly, several approaches are proposed to improve their biological properties. Nucleic acids can be integrated with various types of nanomaterials such as lipid-based nanoparticles, gold nanoparticles, inorganic nanoparticles, or quantum dots to achieve superior performance in biomedical applications [12,13,14]. Alternatively, chemical modifications of natural nucleic acids with altered properties are significant in the development of useful nucleic-acid-based agents [15,16]. Until now, many nucleic acid analogues with modifications at the site of nucleotide backbone, sugar, or bases have been synthesized and investigated. Ideally, the nucleic acid analogues should have certain important chemical or biological properties concerning binding affinity, enzymatic stability, biocompatibility, pharmacokinetics, and biodistribution. In addition, DNA has found widespread use as an attractive building block for constructing self-assembled nanostructures in the field of DNA nanotechnology. Its predictable and programmable Watson–Crick base-pairing properties have been leveraged to create DNA-based nanomaterials with precisely controlled two- or three-dimensional shapes and sizes [17]. Compared with free nucleic acids, DNA assemblies are more reliable biostable, can be better internalized in, and have reduced immunogenicity when rationally designed and modified [18].

**Figure 1:**
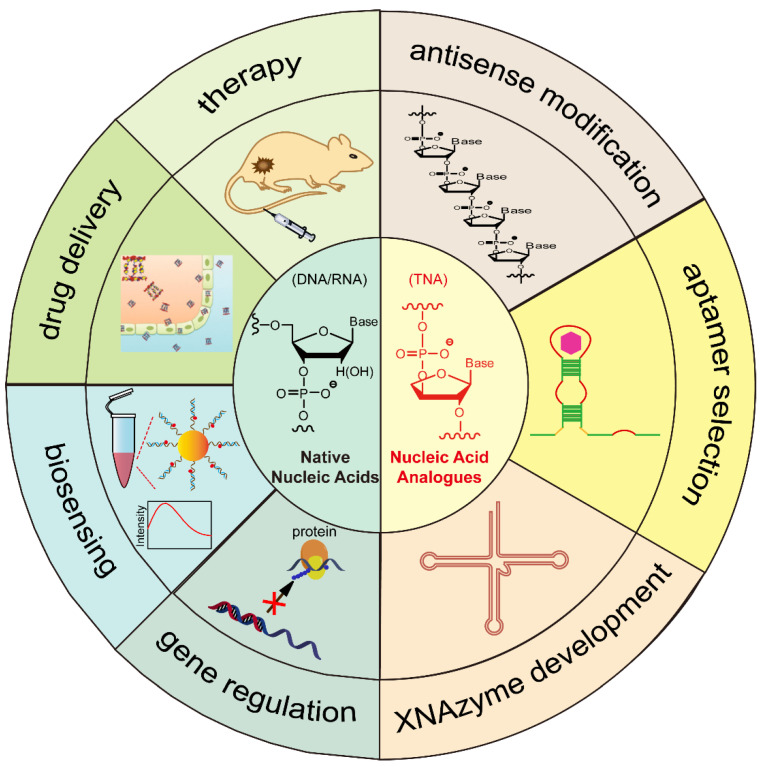
Schematic illustration of nucleic acids and their analogues for biomedical applications.

In this review, we will focus on the development of biomedical applications of structural and functional nucleic acids over the past ten years (Figure 1). In particular, the chemical structures, synthesis, and biomedical applications of native nucleic acids and chemically modified nucleic acid analogues will be discussed and emphasized. Additionally, the recent progress in the development of functional biomaterials based on self-assembled DNA nanostructures for gene regulation, biosensing, drug delivery, and therapy will be presented. This review article will also highlight some of the challenges faced and suggestions for the future development of nucleic-acid-based theranostic reagents for biomedical applications.

## 2. Native Nucleic Acids

### 2.1. Molecular Structure

Nucleic acids are biopolymers composed of a nitrogenous base (adenine (A), guanine (G), cytosine (C), thymine (T), and uracil (U)), that is connected to the 5-carbon sugar ring via an N-glycosidic bond, and 3′ to 5′ phosphodiester bond along the polymer backbone between each nucleotide (Figure 1) [2]. Structurally, RNA is composed of ribose sugar groups with A-U pairs while DNA is composed of deoxyribose sugar groups with A-T pairs. The primary structure of nucleic acids exists as a single-stranded oligonucleotide. Two complementary nucleic acids can form antiparallel duplex structures in aqueous solutions and in living organisms. The molecular structure of the DNA duplex was first revealed by Watson and Crick in 1953, extending biology research to the molecular level [19]. Watson and Crick’s proposed model of the DNA duplex is defined as the B-form. The two DNA/RNA strands with complementary sequences bind to form a duplex that follows the Watson–Crick base-pairing rules: A binds to T (U) with two hydrogen bonds; G binds to C with three hydrogen bonds. The thermal stability of the nucleic acid duplexes is highly dependent on the base composition and number of bases in the strands. A classic study on DNA thermal stability revealed a linear relationship between the relative content of the GC base pairs in the duplexes and the melting temperature (Tm) [20,21]. In addition, the higher-ordered nucleic acid structures are usually constrained by other factors, such as steric crowding, surrounding water molecules, chaperones, and interactions with ions [22,23].

Subsequently, A-form DNA and Z-form DNA that follows the Watson–Crick base-pairing rules have also been discovered and investigated [24]. Besides the Watson–Crick base-pairing rules, the Hoogsteen base-pairing rules have also been described, where base pairs are formed between A and T (U) and between G and protonated C [25]. Unlike Watson–Crick base pairs, Hoogsteen base pairs adopt a syn rather than anti conformation upon the rotation of the purine bases around the glycosidic bond at 180° [26]. Unique nucleic acid structures, such as triplex structures and quadruplex structures, can be constructed based on the Hoogsteen base-pairing rules [22]. For example, the C-rich oligonucleotide is highly sensitive to pH changes. They fold between i-motif structure and the random strand states under appropriate pH values [27]. G-rich DNA strands can form G-quadruplex in the presence of cations and then dissociate by adding the ion-chelating ligands [28].

### 2.2. Synthesis of Nucleic Acids

The chemical synthesis of DNA or RNA is typically carried out via well-established solid-phase phosphoramidite chemistry. At an early stage, DNA synthesis via phosphoramidite technique was realized upon two observations: rapid reacting of chloro- or dichlorophosphites with the 3’-OH group of a 2’-deoxynucleoside, and the synthesis of 2’-deoxythymidine pentanucleotides using dichlorophosphites [29]. These achievements led to the exploration of using 2’-deoxynucleoside P(III) derivatives to synthesize DNA on solid supports; for example, using HPLC-grade silica or controlled pore glass (CPG) [30]. The synthesis is initiated by a detritylation step to remove the 5’-DMT (4,4’-dimethoxytrityl)-protecting group from the nucleoside on CPG. Afterwards, a phosphoramidite monomer as the next nucleotide is added to react with the free 5’-OH of the nucleoside on CPG during the coupling step. Subsequently, the capping step is conducted to deactivate the unreacted 5’-OH groups, preventing their further reaction in the next cycle. The final step is oxidation of the newly formed phosphite triester that is unnatural and unstable. After the chemical synthesis, the polymers can be cleaved from the CPG and purified. The optimized length in chemical synthesis is around 150–200 nt for DNA and 50–60 nt for RNA [29,31].

Longer nucleic acid strands can be obtained via an enzymatic synthesis approach [32]. Conventional DNA polymerases are template-dependent and cannot be used for de novo DNA synthesis, driving the need for template-independent DNA synthases. In early studies, Gilham and coworkers proposed a method for the synthesis of oligonucleotides with well-defined sequences using polynucleotide phosphorylase [33]. Uhlenbeck and coworkers reported enzymatic oligoribonucleotide synthesis using the T4 RNA ligase [34]. The template-independent polymerase terminal deoxynucleotidyl transferase (TdT) is currently the most widely studied enzyme for de novo DNA synthesis. For example, Keasling et al. developed TdT-deoxyribonucleoside triphosphate (dNTP) conjugates and used these molecules for enzymatic de novo synthesis of oligonucleotide DNA with an average yield of 97.7% [31]. For RNA synthesis, in vitro evolution has been employed to select active RNA polymerase ribozyme. For example, Holliger et al. reported an engineered RNA polymerase ribozyme that is capable of RNA synthesis up to 95 nucleotides [35].

### 2.3. Biomedical Applications

#### 2.3.1. Hybridization-Based Applications

**Figure 2:**
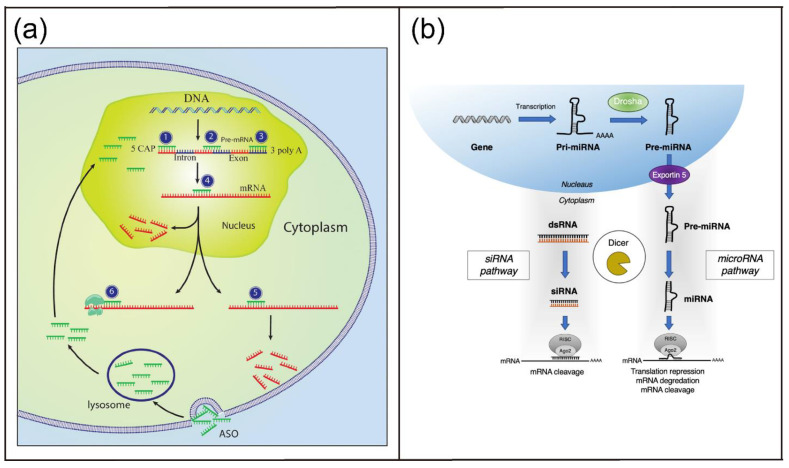
Nucleic acid tools for gene regulation. (**a**) Mechanisms of action of ASOs. Reprinted with permission from [21]. Copyright 2019, Springer Nature. (**b**) Mechanism of RNAi mediated by siRNAs and miRNAs. Reprinted with permission from [22]. Copyright 2020, Wiley-VCH.

The basic function of nucleic acids in molecular biology is gene coding and regulation, which requires base pairing of complementary sequences and the formation of duplex structures. Accordingly, various types of nucleic acids have been used as tools for gene regulation and drugs for the treatment of human diseases with genetic disorders, such as ASOs, siRNAs, and microRNAs (miRNAs) that require base pairing to the complementary sequences (Figure 2) [6]. In general, ASOs refer to synthetic single-stranded oligonucleotide (ON) analogues for which the sequences can hybridize with target messenger RNAs (mRNAs) in a complementary manner (Figure 2a). ASOs can mediate gene regulation via induction of RNase H-dependent degradation of target mRNAs. Alternatively, ASOs can inhibit the protein translation via steric blockage of the target mRNAs [36]. siRNAs and miRNAs mediate gene silencing via RNAi, a biological process of posttranscriptional gene regulation via specific degradation of target mRNA induced by short double-stranded RNA (dsRNA) (Figure 2b) [37]. The most recent and popular gene-editing technique is the CRISPR-Cas9 gene-editing system, information about this gene regulation tool can be found in other reviews [38,39].

Efforts are currently underway to apply these techniques in biomedical research and clinical therapeutics. For example, Cioca et al. reported that a combination of siRNAs complementary to Bcl-2 and c-raf genes can induce apoptosis in HL-60, U937, and THP-1 leukemia cell lines; this combination of siRNA can also enhance the efficacy of therapeutic drugs such as etoposide and daunorubicin [40]. Slack et al. reported the suppression of lung cancer growth by the let-7 family of miRNAs [41]. The introduction of let-7 miRNAs significantly inhibited the growth of various human lung cancer cell lines, as well as the growth of lung cancer xenografts in mice. Additionally, ASO-based therapeutic drugs have been investigated for the treatment of various diseases, including cancer, influenza, neurodegenerative disorders, and hypertriglyceridemia, among others [36].

**Figure 3:**
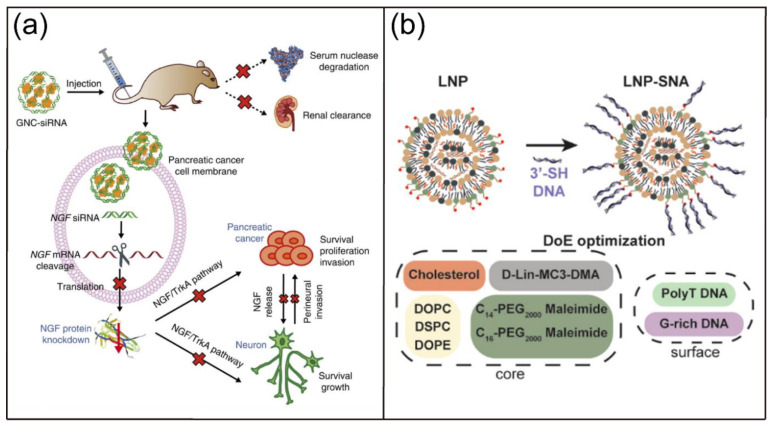
Nanoscale vectors for nucleic acid delivery. (**a**) Schematic illumination of gold nanocluster for delivery of NGF siRNA for gene silencing and pancreatic cancer therapy. Reprinted with permission from [37]. Copyright 2017, Springer Nature. (**b**) Schematic illumination of LNP-SNAs for the delivery of DNA and RNA into the cell cytoplasm. Reprinted with permission from [39]. Copyright 2021, American Chemical Society.

Moreover, various nanoscale vectors, including lipid-based vectors, polymer-based nanocarriers, gold nanoparticles, inorganic nanoparticles, and self-assembled nucleic acid nanostructure, have been developed for efficient and safe therapeutic nucleic acid delivery to address the limitations of natural nucleic acids such as poor biological stability, limited cellular uptake, and unexpected tissue accumulation [7,42,43,44]. For example, Mirkin and coworkers reported the preparation of gold nanoparticle (AuNP)-ON conjugates and their use in intracellular gene regulation [45]. These AuNP-ON conjugates had higher affinity constants for complementary sequences compared with unmodified ON counterparts. Importantly, they showed less susceptibility to nuclease degradation, improved cellular uptake, and were less cytotoxicity. In another study, Jiang and coworkers demonstrated the delivery of siRNA complementary to nerve growth factor (NGF) by gold nanoclusters (GNC-siRNA), which lead to the efficient NGF gene inhibition and pancreatic cancer treatment (Figure 3a) [46]. The GNC-siRNA had increased biological stability, prolonged blood circulation lifetime, and enhanced cellular uptake and accumulation of siRNA in the tumor. The GNC-siRNA efficiently downregulated the NGF expression in Panc-1 cells, pancreatic tumors, and further inhibited the tumor progression in three pancreatic tumor models. Most recently, Sinegra and coworkers synthesized the lipid nanoparticle spherical nucleic acids (LNP-SNAs) for the delivery of DNA and RNA into the cell cytoplasm (Figure 3b) [47]. A library of LNP-SNAs were synthesized via the ethanol dilution approach by mixing DNAs or RNAs in aqueous solution with different lipids including an ionizable lipid, phospholipid, lipid-PEG, and cholesterol in ethanol. The results showed that the optimized LNP-SNAs reduce the required concentration of siRNA for mRNA silencing by two orders of magnitude compared with liposome-based SNAs. Moreover, the LNP-SNAs had altered biodistribution and efficacy profiles in vivo. In addition, Oupický and coworkers reported a mesoporous silica nanoparticle (iMSN)-based multifunctional system for delivery of siRNA and miRNA [48]. In their design, a photosensitizer indocyanine green (ICG) was encapsulated to promote the endosomal escape and a cyclic peptide of 9-amino acids including an Arg-Gly-Asp motif (iRGD) was conjugated on the surface to achieve deeper tumor penetration. Intravenous administration of the iMSNs loaded with siPlk1 and miR-200c led to remarkable suppression of the primary tumor growth and significant reduction of cancer metastasis upon irradiation with lights.

Due to their specificity and flexibility, hybridization-based probes have also been designed based on the Watson–Crick base-pairing rules to detect complementary targets, such as DNA, mRNA, miRNA, and non-coding RNA [49,50]. For example, linear antisense probes labeled with a fluorophore can be used to detect their complementary DNA or RNA sequences demonstrating their capability in intracellular RNA detection and imaging [50]. In addition, nanoparticles are widely interfaced with nucleic acids for sensing and detection applications because of the enhanced signal, excellent biological stability, and self-delivery capability in cells. For example, Mirkin’s group reported the development of nano-flares that consist of a dense shell of duplex DNA on AuNPs [51] where binding by the recognition strand to the target mRNA resulted in enhanced fluorescence.

#### 2.3.2. Catalysis-Based Applications

Besides carrying, transferring, and expressing genetic information, some functional RNA molecules are able to catalyze certain biochemical reactions; they are called “ribozymes” [4]. Catalytic functions of ribozymes can be generally categorized into three groups: cleavage, splicing, and others. So far, many novel artificial ribozymes with various unanticipated but interesting properties have been obtained by in vitro selection techniques [52]. Following the discovery of ribozymes, DNA-based enzymes, called DNAzymes, were also obtained by the same selection techniques. In general, a diverse library (composed of ∼10^14^ molecules) of nucleic acids with random sequences is subjected to a series of selection steps for separation of the target strands with catalytic functions. With several rounds of selection, isolation, and amplification, diverse ribozymes with high affinity and selectivity for target molecules can be gradually enriched and isolated [53].

**Figure 4:**
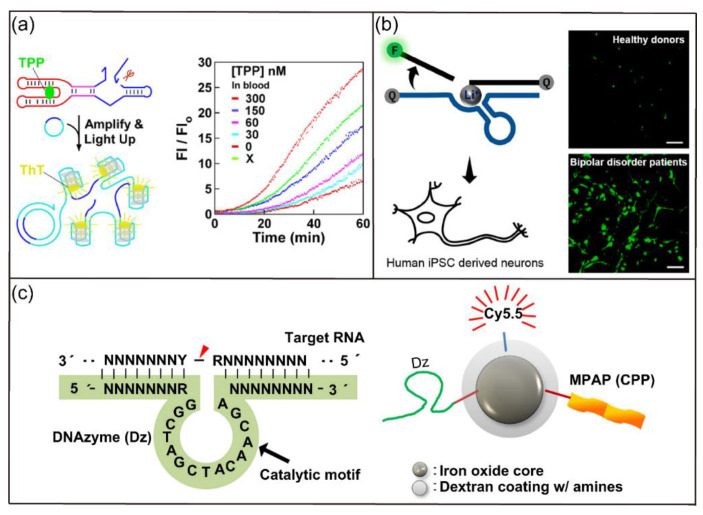
Biomedical applications of ribozymes and DNAzymes. (**a**) Schematic illumination of allosteric ribozymes for detection of TPP in whole blood. Reprinted with permission from [47]. Copyright 2021, American Chemical Society. (**b**) Schematic illumination of Li^+^-specific DNAzyme sensor for fluorescent imaging of Li^+^ in living cells. Reprinted with permission from [48]. Copyright 2021, American Chemical Society. (**c**) Schematic illumination of iron oxide nanoparticle-based delivery of DNAzyme for HCV gene suppression. Reprinted with permission from [50]. Copyright 2012, Elsevier.

Based on their catalysis property, ribozymes and DNAzymes have been used in the development of sensing platforms for detecting ions and molecules. For example, Liu and coworkers reported a fluorescent biosensor based on a graphene-DNAzyme catalytic beacon for detection of Cu^2+^ [54]. The graphene functioned as the scaffold and the quencher of the Cu^2+^-dependent DNAzyme. Cu^2+^-induced cleavage of the DNA, however, destabilized the graphene-DNAzyme complex, resulting in fluorescence recovery. The developed biosensor had greater sensitivity than common DNAzyme-based sensors. Recently, Du and colleagues reported a ribozyme-based biosensor for quantitative measurement of thiamine pyrophosphate (TPP) in whole blood samples (Figure 4a) [55]. The authors isolated an allosteric ribozyme for TPP and developed a novel blood sample preparation protocol applicable for RNA detection. They then demonstrated the fluorescence detection of TPP in whole blood via the mix-and-read operation. In addition, McGhee et al. reported the selection and development of a Li^+^-specific DNAzyme sensor with >100-fold selectivity compared with other biorelevant metal ions [56]. As shown in Figure 4b, the fluorescent sensor is designed for imaging of Li^+^ in HeLa cells, human neuronal progenitor cells, and neurons. Strikingly, the biosensor was able to differentiate the significant increase of Li^+^ accumulation in differentiated neurons derived from bipolar disorder patients compared with healthy controls.

Ribozymes and DNAzymes have also been used widely in therapeutic gene regulation applications [57]. For example, Ryoo et al. reported a DNAzyme delivery system based on nanoparticles for therapeutic suppression of hepatitis C virus (HCV) NS3 gene and treatment of hepatitis C (Figure 4c) [58]. The developed nanocomplex can be used to silence target NS3 without inducing significant cytotoxicity. In addition, the nanocomplex could specifically accumulate in hepatocytes, indicating its applicability in the treatment of hepatitis C. In another study, Rouge and coworkers reported a strategy to prepare SNA architecture to stabilize and deliver ribozymes into live cells [59]. They reported that the ribozyme-SNA complex induced the cleavage of O^6^-methylguanine-DNA methyltransferase (MGMT) mRNA, downregulated MGMT protein, and increased sensitization of glioblastoma multiforme (GBM) cells to chemotherapy.

#### 2.3.3. Binding-Activity-Based Applications

**Figure 5:**
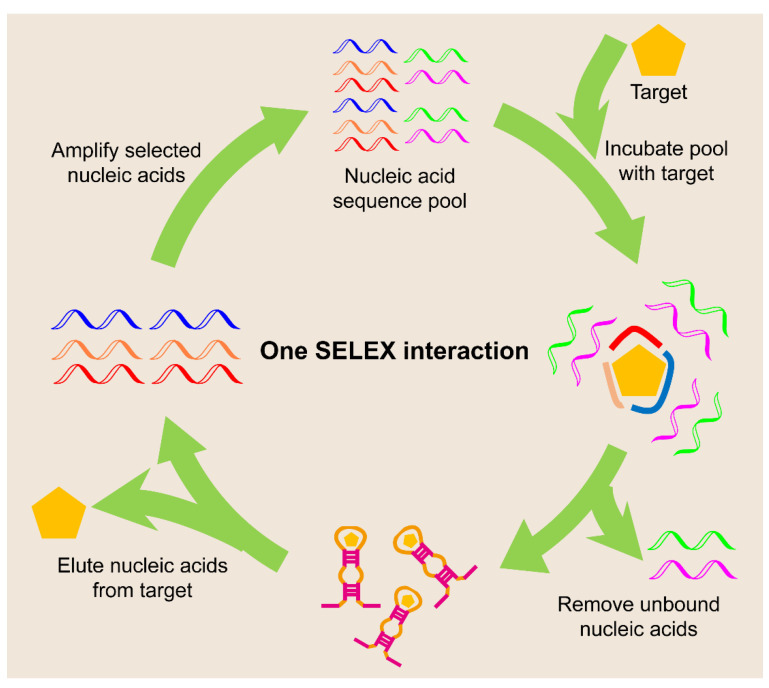
The schematic illumination of the SELEX process.

Aptamers are another class of functional single-stranded nucleic acids that bind to various chemical and biological targets, such as small molecules, biomacromolecules, or even whole cells, with high specificity and affinity [60]. Aptamers are usually obtained via an in vitro selection method called systematic evolution of ligands by exponential enrichment (SELEX, Figure 5). This technique involves repeated rounds of binding, selection, and amplification from a pool of nucleic acid molecules with diversity (~10^14^ distinct sequences) in order to progressively separate the target single-stranded nucleic acids with certain functions, such as target binding or catalysis [61].

Aptamer-based sensors (aptasensors) have been extensively developed for detecting ions, small molecules, proteins, and cells because of several unique properties; high thermal and physiological stability, low immunotoxicity, simple chemical synthesis, and modifications [13,62,63]. For example, Fan et al. designed a DNA probe for anti-adenosine triphosphate (ATP) detection based on an anti-ATP aptamer tagged with ferrocene and the complementary DNA strand [64]. In the presence of ATP, the complementary strand was displaced while the aptamer folded into a 3D structure. This brought the ferrocene tag close to the electrode, resulting in a signal change. Wu and coworkers designed a graphene field-effect transistor functionalized with pyrene-tagged DNA aptamers for sensitive detection of *Escherichia coli* (*E. coli*) [65]. The binding of the negatively charged *E. coli* resulted in the conformational change of the DNA aptamer where the pyrene group was much closer to the graphene surface, leading to an increment in the hole carrier density in graphene. These developed electrical biosensors have excellent sensitivity, selectivity, and stability. In addition, our group successfully developed a versatile detection platform via integration of three different aptamers along the edges of DNA nanotubes [66]. The developed aptasensor were highly sensitive and, simultaneously detected three targets such as thrombin, ATP, and insulin molecules. These DNA-based nanoplatforms allow the combination of multiple binding activities into one single system, enabling the spatial orientation of various targets for detection in complex environments.

Aptamers for disease diagnostics and therapeutics, especially for cancer, have also been reported [67]. For example, Xian and coworkers developed an aptasensor based on near-infrared fluorescent Ag_2_S nanodots and immune-magnetic spheres (MNs) for capture and detection of circulating tumor cells (CTCs). This development has great potential in cancer diagnostics and therapeutics [68]. In addition, Ding et al. developed a DNA robot for targeted thrombin delivery [69]. A nucleolin-targeting DNA aptamer was functionalized on the outside of a DNA robot that served as a targeting domain and a molecular trigger for mechanical opening of the DNA nanorobot. After intravenous injection, the DNA nanorobot opened itself upon recognition of the targeted blood vessel surface and released thrombin, inducing intravascular thrombosis and subsequent tumor growth suppression. Most recently, Tan and coworkers developed a bifunctional aptamer to improve the blood-brain barrier (BBB) penetration for enhanced tauopathy therapy [70]. The bifunctional aptamer was composed of a transferrin receptor (TfR) aptamer and a Tau protein aptamer. The TfR aptamer recognized endothelial cells for transcytosis into the cell. The Tau aptamer inhibited Tau phosphorylation and other pathological processes related to tauopathy in the brain. It is envision that the developed bifunctional aptamer demonstrated the ability to effectively reduce the traumatic brain injury (TBI)-related biomarker levels and to improve the impaired memory restoration.

## 3. Chemically Modified Nucleic Acid Analogues

To improve the biological properties of nucleic acids, chemically modified nucleic acids called nucleic acid analogues have been proposed for the development of useful nucleic acid agents in the areas of biological and biomedical applications. Nucleic acid analogues should ideally possess improved biological properties compared with natural DNA/RNA such as increased nuclease resistance, increase binding affinity to the complementary strand, reduced immune responses, and enhanced cell penetration or tissue specificity [15,71].

**Figure 6:**
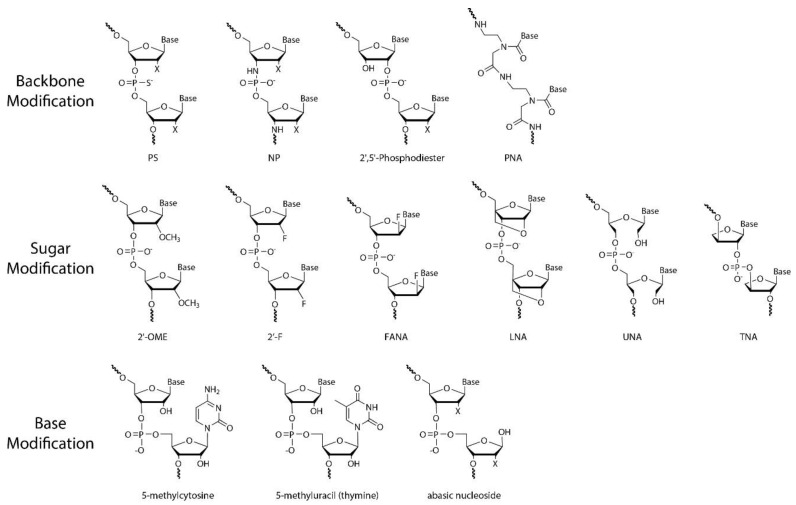
Selected examples of nucleic acid analogues with modifications of backbone, sugar, and base. PS: phosphorothioate; NP: N3‘ → P5′ phosphoramidate; PNA: peptide nucleic acid; 2′-OME: 2′-O-methyl RNA; 2′-F: 2′-deoxy-2′-fluoro RNA; FANA: 2′-deoxy-2′-fluoroarabinonucleic acid; LNA: locked nucleic acid; UNA: unlocked nucleic acid; TNA: threose nucleic acid.

As discussed in Section 2, a nucleotide has three subunits: phosphodiester linkage, a nucleobase, and a sugar moiety. As shown in Figure 6, the chemical modifications can be introduced at the site of the phosphodiester backbone, sugar moiety, nucleobase, or combined sites thereof [71,72]. Specifically, nucleic acid analogues with chemical modifications on the sugar moiety are called xeno-nucleic acids (XNAs) [73]. We will briefly introduce several XNAs in this review (Table 1).

### 3.1. Phosphorothioate (PS) ONs

#### 3.1.1. Molecular Structure of PS ONs

Phosphorothioate (PS) ONs refer to nucleic acids with a modified phosphate backbone, in which one of the non-bridging oxygen atoms is replaced with sulfur. The PS modification can be achieved in both DNA and RNA.

The PS modification offers PS ONs interesting structural and biochemical properties. It does not change the B-form conformation; and PS-modified DNA can still form duplex structures with DNA or RNA. In addition, the PS modification dramatically increases the nuclease resistance of the PS ONs. The half-life of PS-modified DNA (>24 h) in physiological conditions is much longer compared with natural DNA (15–60 min), leading to improved in vivo pharmacokinetics [74]. Particularly, PS ONs can enter cells without additional modification or formulation. They are capable of recruiting RNase H, leading to sequence-specific cleavage of different RNA targets [75]. PS modification leads to a relatively unstable duplex because duplexes with PS modifications have lower Tm values than the corresponding duplexes without modification [74].

#### 3.1.2. Synthesis of PS ONs

In 1967, Eckstein et al. first reported a dinucleotide with PS linkage that had strong enzymatic resistance. This initial finding led to increased interest in ON sulfurization [76]. Subsequently, a few techniques for chemical synthesis of dinucleoside phosphorothioates have been reported. The most favorable technique is based on the phosphite triester for PS ON synthesis where the oxidation step is replaced by oxidative sulfurization [76]. Historically, elemental sulfur was the first sulfurizing reagent reported for solid-phase synthesis of PS ONs [77]. Currently, 3-((dimethylamino-methylidene)amino)-3H-1,2,4-dithiazole-3-thione (DDTT) is the most widely used sulfuring reagent for the synthesis of PS ONs due to its low cost, high efficacy, good stability, and excellent RNA sulfurization [77]. At present, PS ONs can be synthesized easily using a DNA synthesizer via the well-established phosphoramidite chemistry. Thus, PS ONs are now commercially available.

In addition, enzymatic synthesis of PS ONs has been studied by using chiral PS nucleotides as substrates. For example, DNA polymerase I enables the polymerization of (Sp)-dATPαS on a poly(dT) template. Interestingly, dNTPαS displays normal base-pairing properties. Up until now, more than 50 enzymes have been investigated for catalysis of nucleotidyl transfer [76].

#### 3.1.3. Biomedical Applications of PS ONs

Of note, the PS backbone modification was first used in antisense application and is still the most widely used modification in nucleic-acid-based therapeutics [16]. For example, fomivirsen, the first ASO antiviral drug approved by the U.S. FDA, is a fully 21-mer PS-modified DNA ON. Fomivirsen significantly delays the progression of cytomegalovirus (CMV) retinitis in immunocompromised patients by inhibiting viral replication by directly targeting IE2 mRNAs thus suppressing the expression of IE2 proteins. There are also PS-modified ASO drugs in clinical trials. Genasense is, such example, where it is PS-modified ASOs targeting anti-apoptotic gene Bcl-2 mRNAs that inhibits Bcl-2 mRNA translation and expression. Another example is custirsen, which is a PS gapmer ASO targeting clusterin with a chemosensitizing property [6].

PS modification has also been applied to siRNA and miRNA strands to confer nuclease resistance [71]. For example, Braasch and coworkers tested the effects of PS modification on RNA stability and inhibition of gene expression [78]. They found that PS-modified RNA duplexes are remarkably stable in serum. Treatment of cells with RNA duplexes bearing PS modification led to selective inhibition of gene expression. Recently, our group reported a nanodiamond (ND)-based platform for targeted nuclear delivery of ANA4625 PS-modified ASO [79]. The ND was coated with human immunodeficiency virus TAT protein and a nuclear localization signal (NLS) peptide. The TAT-NLS-NDs had low cytotoxicity, high affinity to ANA4625, and enhanced cellular uptake. The ANA4625-TAT-NLS-NDs had enhanced therapeutic efficacy by inhibiting Bcl-2 and Bcl-xL expression and inducing cancer cell apoptosis.

PS modification has been used to select aptamers and enzymes with enhanced physiological stability. For example, King et al. developed a novel combinatorial approach to construct and select PS-modified DNA aptamers with enhanced nuclease resistance [80]. Aptamers that specifically bind to the target nuclear factor for human IL6 (NF-IL6) was e obtained by using SELEX from a random library of 22-nucleotide-long duplexes bearing one or multiple nucleotides with α-thio-(d)NTPs. In addition, Abeydeera and coworkers developed an α-thrombin-targeting RNA aptamer bearing the phosphorodithioate substitution on a single nucleotide with a dramatic 1000-fold improved target binding affinity [81]. Most recently, Liu and coworkers designed a sensor based on the Ce13d DNAzyme that can cleave the PS-modified substrate in the presence of thiophilic metal ions [74]. By labeling the substrate strand with a fluorophore and the enzyme strand with a quencher, the sensor showed a fluorescence response in the presence of various metal ions.

### 3.2. Peptide Nucleic Acid (PNA)

#### 3.2.1. Molecular Structure of PNA

Peptide nucleic acid (PNA), which was first invented by Nielsen et al., is one of the most widely investigated nucleic acid analogues [71,82,83]. PNA is a synthetic nucleic acid analogue in which the sugar-phosphate backbone is replaced with a peptidic backbone. Therefore, PNA possesses the properties of both peptides and nucleic acids.

In PNA, the backbone consists of the N-(2-aminoethyl) glycine repeating units, and the polyamide chain is linked covalently to nucleobases through a carboxymethyl spacer. PNA can bind to the complementary DNA and RNA strands through Watson–Crick base-pairing rules due to the natural nucleobases in PNA [84]. The PNA/DNA or PNA/RNA duplexes exhibit stronger binding affinity and sequence specificity than DNA and RNA duplexes due to the absent electrostatic repulsion in a neutral backbone. Consequently, the thermal stability of PNA/DNA or PNA/RNA duplexes is relatively higher. Besides, PNA can bind to DNA/RNA via Hoogsteen base pairing rules, leading to the formation of triplex structures [85]. In addition to the excellent chemical stability, PNA possesses excellent biological stability because the complex structure of PNA gives rise to strong resistance to enzymes. Nevertheless, PNA shows some drawbacks including low aqueous solubility and the propensity to self-aggregate. Thus, various chemical alterations in the PNA backbone have been implemented to improve its properties and to make it a better tool for diverse applications [86,87].

#### 3.2.2. Synthesis of PNA

The synthesis of PNA oligomers or polymers is virtually identical to the synthesis of peptides [88]. Thus, PNA can be easily prepared via well-established automated solid-phase synthesis. The elongation of PNAs occurs via the condensation between the carboxyl group of the building block and the deprotected amino group of the growing chain. During synthesis, the amino groups of PNA monomers are protected by Bhoc or Fmoc moieties. Specifically, the Bhoc group protects the exocyclic monomers of A, G, C, and T while the Fmoc group protects the primary amino acids in the monomer backbone. At the end of the synthesis, the Bhoc group is removed by trifluoroacetic acid, while the Fmoc group is removed by a solution of 20% piperidine in dimethylformamide.

In addition, manual synthesis of PNA based on commercially available or self-made building blocks is also employed for efficient synthesis of large-scale PNA; however it is a time-consuming process [89]. The aminoethyl glycine backbone is assembled via alkylation of a protected ethylenediamine or reductive amination of N-protected aminoacetaldehyde. The protected base components are then converted into N-alkyl acetic acid derivatives. The backbone and the base components are then linked via the formation of an amide bond.

#### 3.2.3. Biomedical Applications of PNA

PNAs have been widely applied in antisense technology due to the metabolic stability and ability to bind mRNA. In principle, PNAs sterically block the initiation site and inhibit mRNA splicing or translation [86]. Ly and coworkers reported the antisense application of cell-permeable, guanidine-based PNA (GPNA) [90]. It bound to the transcriptional initiation site of human E-cadherin gene inducing potent and specific antisense activities. In addition, GPNA was less cytotoxic compared with PNA-polyarginine conjugates. In a recent study, Bruchez et al. demonstrated the reversible suppression of a luciferase gene using a γ-modified PNA (γPNA) sequence and a non-complementary toehold [91]. The antisense γPNA strand could be removed by a second, fully complementary γPNA strand via a strand displacement reaction, leading to the continuation of translation. In addition to the development of PNA-based antisense agents, delivery platforms have also been investigated for selective delivery of PNA to targeted cells to achieve improved antisense efficacy in vivo [92,93].

PNAs have great promise for the development of fluorescence in situ hybridization (FISH) probes. For example, Chen et al. developed short PNA oligomer probes for the detection of chromosomal abnormalities and repeat structure in the human genome [94]. In total, 10 PNA probes specific for human chromosomes 1, 2, 7, 9, 11, 17, 18, X, and Y have been designed. These chromosome-specific PNA probes were able to detect simple aneuploidies in humans, while other PNA-based probes could also be used for chromosome “bar-coding” as sequence-specific stains. In addition, Machado and coworkers developed a novel PNA FISH probe (Lac663 PNA probe) for accurate Lactobacillus spp. identification [95]. The Lac663 PNA probe was tested on 36 strains of Lactobacillus species and 20 strains of other bacterial species. The sensitivity and specificity were found to be 100% and 95%, respectively. In addition, the Lac663 PNA probe could detect Lactobacillus spp. in fresh milk samples with an addition of Lactobacillus strains at concentrations found in probiotics as well as other taxonomically related bacteria and pathogenic bacteria.

**Figure 7:**
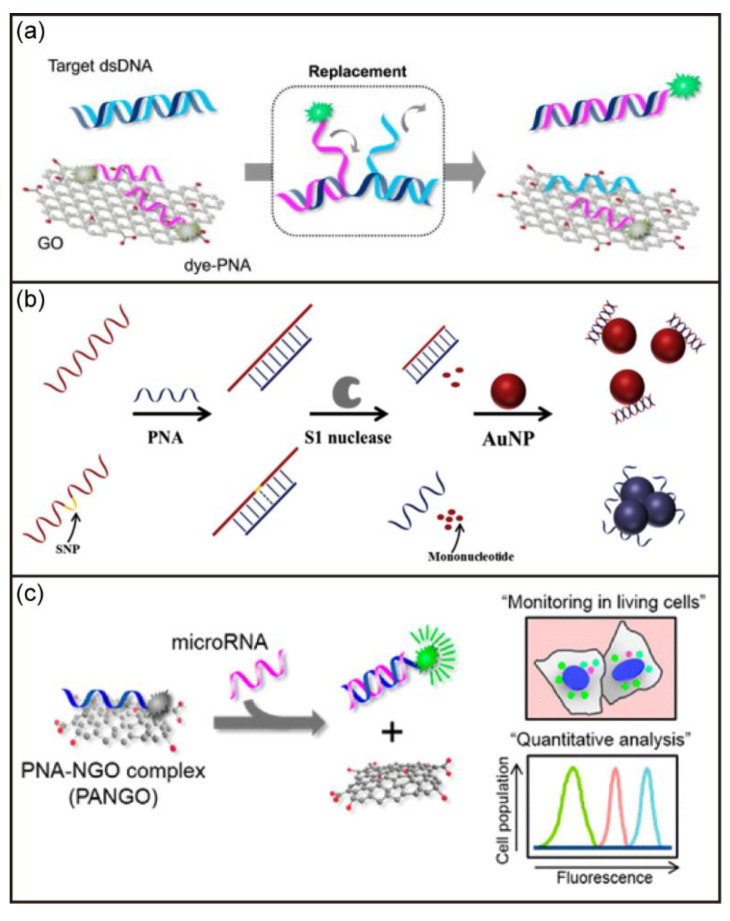
PNA-based biosensors. (**a**) Schematic illumination of dsDNA detection using PNA and graphene oxide. Reprinted with permission from [96]. Copyright 2014, Elsevier. (**b**) Schematic illustration of single-base mismatch detection using PNA and AuNPs. Reprinted with permission from [97]. Copyright 2019, Elsevier. (**c**) Schematic illustration of multiplexed microRNA sensing using PNA and nano graphene oxide. Reprinted with permission from [99]. Copyright 2013, American Chemical Society.

The high sensitivity and specificity to DNA/RNA have also led to the rapid development of PNA-based biosensors. In 1996, Wang et al. reported a PNA-based electrochemical biosensor for the first time [96]. A 15-mer PNA probe was attached onto a carbon-paste electrode transducer to detect PNA/DNA duplex formation. The PNA-based electrochemical biosensor had high sensitivity and specificity, fast detection of targets even at room temperature, and minimal dependence on ionic strengths. Subsequently, Lee et al. developed a sensitive DNA biosensor based on the quenching effect of graphene oxide and the fluorescent dye in the PNA sequence (Figure 7a) [97]. Various DNA targets could be detected by simply changing the PNA sequence. Xing and coworkers also reported a label-free biosensor using PNA as capture probes for simple and fast detection of target DNAs [98]. As shown in Figure 7b, this method utilized the capability of PNA to distinguish DNA with some mismatches. The exonuclease could cut the DNA into fragments when PNA was bound to DNA with mismatches. In contrast, DNA could be protected from degradation when completely complementary to PNA. Together with the aggregation-inducing effect of PNA on AuNPs, colorimetric analysis of the target DNA can be achieved. In addition, Min and coworkers demonstrated multiplexed miRNA sensing based on dye-labeled PNA and nano graphene oxide (Figure 7c) [99]. The developed biosensors indicated the fluorescent dye of labeled PNA was quenched but it was recovered upon addition of the target miRNAs. The biosensor allowed simultaneous detection of three different target miRNAs with a detection limit of about 1 pM.

### 3.3. Sugar 2′-O-Methyl (2′-OMe) RNA

#### 3.3.1. Molecular Structure of 2′-OMe RNA

Besides the backbone modifications, modifications on the sugar moiety, specifically named XNAs, are a common type of alteration to nucleic acids. For example, the ribose sugar can be modified by replacing 2′-hydroxyl with other chemical groups. The 2′-O-Methyl (2′-OMe) RNA, in which a methyl group is added to the 2′-hydroxyl of the native ribose, is one of the most widely used sugar modifications [100].

The 2′-OMe-modified sugar adopts a conformation like RNA and forms A-form duplexes, suggesting that such a unit is particularly well tolerated in RNA modification [101]. 2′-OMe-modified RNA can form duplexes with complementary DNA and RNA strands. The incorporation of 2′-OMe modifications into RNA enhances the binding affinity toward complementary RNA strands compared with DNA. In addition, 2′-OMe modifications significantly increase the nuclease stability of nucleic acids. In one example, RNAs with full 2′-OMe modification were stable after incubation for 8 h in the rat gastrointestinal tract [102]. 2′-OMe modification naturally occurs in biological systems. Thus there are no biosafety concerns regarding this type of modification. Indeed, the 2′-OMe modification was reported to reduce immunostimulatory effects [103].

#### 3.3.2. Synthesis of 2′-OMe RNA

In 1987, Ohtsuka et al. reported the synthesis of 2′-OMe RNA with 2′-O-methyl ribonucleosides and investigated their thermal stability [104]. The 2′-O-methyl ribonucleosides of A, T, and C were synthesized via 2′-O-methylation of protected nucleosides with CH_3_I in the presence of Ag_2_O. On the other hand, the 2′-O-methyl ribonucleosides of G were synthesized through monomethylation of a 2′,3′-cis-diol system with diazomethane. These 2′-O-methyl ribonucleosides were then converted to protected 2′-O-methyl ribonucleoside-3′-phosphates =that were used for solid-phase synthesis of 2′-OMe RNA [104]. Wagner et al. also reported the simple preparation of protected 2′-O-methyl ribonucleoside-3′-O-phosphoramidites via alkylation of the ribonucleosides [105]. In addition, gapmers comprising 2′-OMe nucleotides with natural or chemically modified DNA monomers have been synthesized and investigated [106]. At present, 2′-O-methylphosphoramidites are commercially available for the synthesis of 2′-OMe-modified RNAs via solid-phase synthesis.

Native polymerases do not accept 2′-OMe nucleotide triphosphates (NTPs) as substrates. Accordingly, various genetically modified polymerases have been identified to catalyze the incorporation of 2′-OMe nucleotides into strands. In one study, Fa and coworkers developed an activity-based selection technique to isolate polymerase variants with desirable properties [107]. Based on this method, they successfully isolated one evolved polymerase that can efficiently synthesize 2′-OMe polymers with similar fidelity compared to the natural enzyme with natural substrates. In addition, Chen et al. reported a polymerase-evolution technique and selection of thermostable polymerases to efficiently interconvert 2′-OMe ONs and DNA counterparts via transcription and reverse transcription [108]. The evolved polymerases could also amplify of partially 2′-OMe-modified ONs via the polymerase chain reaction (PCR). Recently, Kestemont and coworkers reported a T4 DNA ligase that is capable of ligating 2′-OMe RNA duplexes and other chemically modified sequences [109].

#### 3.3.3. Biomedical Applications of 2′-OMe RNA

The 2′-OMe modification has been widely investigated in gene regulation and aptamer development research. The FDA approval in 2004 of a therapeutic RNA aptamer, Macugen, having 2′-OMe and 2′-fluoro modifications made this modified nucleic acid analogue more promising. Macugen was isolated via the SELEX process, based on its ability to bind VEGF. It has been used to treat age-related macular degeneration [110]. In addition, fitusiran and inclisiran target the antithrombin gene and PCSK9 gene for the treatment of hemophilia and hypercholesterolemia, respectively. These two 2′-OMe-modified RNA-based drugs are currently in clinical trials [16].

Initial studies showed that the partially 2′-OMe-modified siRNA can maintain RNAi activity and has improved serum stability. For example, Jackson et al. demonstrated that 2′-OMe modifications have potential applications in developing chemically modified siRNA drugs with reduced off-target effects. 2′-OMe modifications at designated sites in siRNA guide strands reduced the gene silencing effect of partially matched mRNA transcripts while not affecting completely complementary targets [111]. Interestingly, Baker and coworkers also reported that siRNAs with full 2′-OMe modifications within sense strands had Argonaute2/eIF2C2-dependent activity, suggesting the potential development of fully 2′-OMe-modified siRNA as functional drugs [112].

Aptamers with 2′-OMe modifications have also been developed and investigated. By using the SELEX process in conjunction with post-SELEX modifications, Green and coworkers identified a 2′-OMe-containing aptamer that binds to vascular endothelial growth factor (VEGF) with high specificity and affinity and enhanced nuclease stability [113]. In another study, Paula et al. demonstrated direct isolation of a 2′-OMe aptamer via the SELEX process only [102]. The authors first identified the transcription conditions to directly generate the 2′-OMe transcripts then used them to isolate a fully 2′-OMe aptamer for VEGF binding.

### 3.4. Sugar 2′-Deoxy-2′-Fluoro (2′-F) RNA

#### 3.4.1. Molecular Structure of 2′-F RNA

The 2′-deoxy-2′-fluoro (2′-F) modification, an analogue of RNA in which the 2′-hydroxyl on the sugar is replaced by fluorine, is another widely studied and used chemical modification.

The 2′-F modification is the best mimic of the 2′-hydroxyl group based on size and charge [16]. 2′-F RNA can form duplex structures with complementary DNA and RNA. In particular, the 2′-F modification significantly increases the binding affinity to complementary RNA sequences with a melting temperature (Tm) increase of 2 to 3 °C per modified nucleotide.2′-F RNA does not have improved nuclease resistance. Consequently, other types of chemical modifications are usually introduced to 2′-F RNA molecules to enhance its nuclease stability. Although 2′-F RNA/RNA duplexes are not substrates of RNase H [103], 2′-F RNA can modulate alternative splicing of target transcripts via the recruitment of the interleukin enhancer-binding factor 2 and 3 complex [114]. In addition, the 2′-F modification can inhibit the innate immune response of siRNA where the inhibitory activity is dependent on the position and number of the incorporated nucleotides [103].

#### 3.4.2. Synthesis of 2′-F RNA

The syntheses of 2′-fluoroadenosine (2′-F-A), 2′-fluoroguanosine (2′-F-G), 2′-fluorouridine (2′-F-U), and 2′-fluorocytidine (2′-F-C) have been reported by different groups [115]. In 1984, Sinha and coworkers reported the synthesis of 2-deoxy-2′-fluoronucleoside 3′-phosphorarnidites that enable automated synthesis of 2′-F RNA [116]. Afterward, Eckstein et al. reported the synthesis of 2′-F-A starting from adenosine with an improved yield of 46% [117]. 2′-F-A was converted into phosphoramidite and further incorporated into a hammerhead ribozyme RNA via automated chemical synthesis. The same group has also reported introduction of 2′-F-C, 2′-F-U, and 2′-F-G into the hammerhead ribozyme RNA [115,117]. Subsequently, Cook et al. reported modified synthetic protocols for all four 2′-F RNA phosphoramidites with improved yields [115]. The chemical synthesis of 2′-F RNA was subsequently described where methanolic ammonia treatment at room temperature was used to deprotect the ONs to avoid the elimination of fluoride. At present, 2′-F RNA phosphoramidites are commercially available for the chemical synthesis of 2′-F RNA via solid-phase synthesis on a DNA synthesizer.

In addition, 2′-F RNA can be synthesized enzymatically. Currently, the T7 RNA polymerase in which tyrosine 639 is replaced with phenylalanine (Y639F) from bacteriophage is the commercially available enzyme for the synthesis of 2′-F RNA [118]. Other polymerases have also been reported. For example, Smith and coworkers reported methods for polymerase-directed synthesis of 2′-F-modified DNA and identified four thermostable DNA polymerases capable of incorporating 2′-fluoronucleotide triphosphates with high efficiency [119]. In another study, Holliger and coworkers identified a Tgo DNA polymerase mutant (E664K), in conjunction with Y409G mutation that is used to synthesize fully pseudouridine, 5-methyl-C, 2′-F, or 2′-azido-modified RNA molecules [120]. Recently, Zhu et al. reported that wild-type Syn5 RNA polymerase had relatively low discrimination against 2′-F-dNTPs compared with that of T7 RNA polymerase [121]. The presence of both Mg^2+^ and Mn^2+^ can reduce this discrimination while retaining the reaction activity. In addition, a mutated Syn5 RNA polymerase, in which tyrosine 564 is replaced with phenylalanine (Y564F), was shown to further reduce the discrimination during RNA synthesis.

#### 3.4.3. Biomedical Applications of 2′-F RNA

2′-F RNA has been widely used in gene regulation and aptamer development due to strong binding affinity to target RNA, high thermal stability, and innate immune response inhibition. Furthermore, the 2′-F modification can also be found in the Macugen, which is an FDA-approved RNA aptamer [110]. At present, revusiran and inclisiran target transthyretin and PCSK9 gene for the treatment of hereditary ATTR amyloidosis and hypercholesterolemia, respectively. These two 2′-F-modified siRNA-based drugs are currently in clinical trials [16].

The 2′-F modification is well tolerated in siRNA in either guide or passenger strands. In an early study, the 2′-F modification was incorporated in a hammerhead ribozyme [117]. The presence of 2′-FU and 2′-FC did not significantly reduce the catalytic efficiency. Moreover, the 2′-F modified ribozyme at all uridine and cytidine sites displayed strong nuclease stability. In another study, Manoharan and coworkers reported that the 2′-F-modified siRNA which targets factor VII mRNA, had increased nuclease stability, decreased immune stimulation in vitro, and improved activity compared with unmodified RNA [122]. Recently, Rigo and coworkers demonstrated that 2′-F-modified ASOs can modulate alternative splicing of target mRNAs, thus offering another approach to regulate gene expression for therapeutic applications [114].

**Figure 8:**
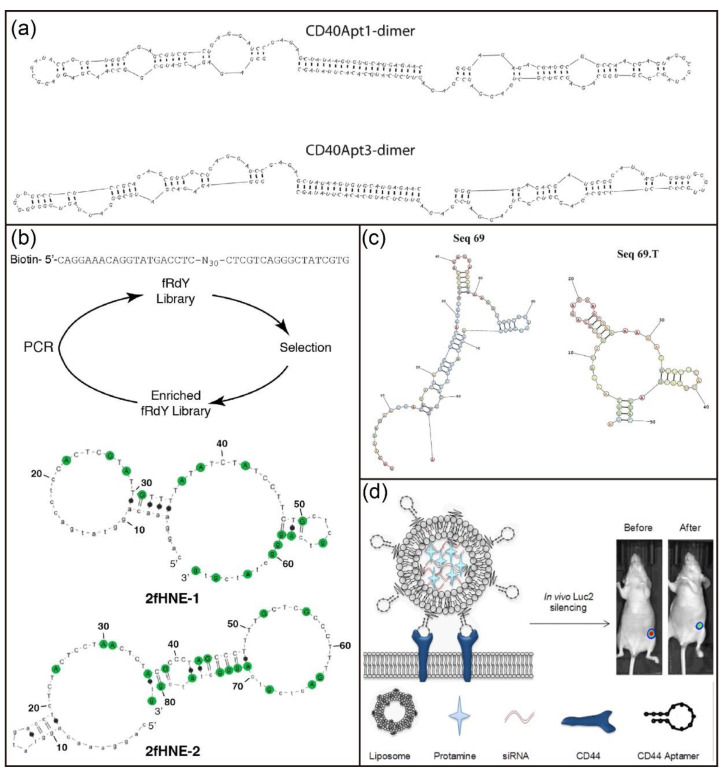
Applications of 2′-F RNA aptamers. (**a**) Predicted secondary structures of CD40Apt1-dimer and CD40Apt3-dimer. Reprinted with permission from [125]. Copyright 2015, Elsevier. (**b**) Selection scheme and predicted secondary structures of 2fHNE-1 and 2fHNE-2 aptamers. Reprinted with permission from [126]. Copyright 2017, American Chemical Society. (**c**) Predicted secondary structures of apt69 full-length and apt69.T aptamers. Reprinted with permission from [127]. Copyright 2019, Elsevier. (**d**) Aptamer-guided nanoplatforms for gene silencing in CD44-expressing tumor model. Reprinted with permission from [130]. Copyright 2018, Elsevier.

The 2′-F modification has also been widely used to develop novel aptamers for molecular recognition and disease therapy. For example, Pagratis et al. investigated 2′-amino-modified and 2′-F-modified aptamers in separate selections against keratinocyte growth factor [123]. The 2′-F modification had higher affinity and bioactivity compared with the 2′-amino modification. Recently, Soldevilla and coworkers identified 2′-F RNA aptamers targeting the CD40 receptor with high affinity via the HS-SELEX technique (Figure 8a) [124]. They further engineered three therapeutic CD40-aptamer-based constructs for the treatment of B-cell lymphoma and bone-marrow aplasia. Thirunavukarasu et al. also reported the isolation of two 2′-F-modified aptamers (2fHNE-1 and 2fHNE-2) that bind human neutrophil elastase (HNE) with high affinity (Figure 8b) [125]. Interestingly, the 2′-F-modified aptamers had strong nuclease resistance, specific interactions with HNE, and negligible nonspecific electrostatic interactions. Recently, Catuogno and coworkers reported the selection of a 2′-F RNA aptamer (apt69.T) that selectively binds to B cell maturation antigen (BCMA) via modified cell-based SELEX (Figure 8c) [126]. Importantly, the developed 2′-F-modified aptamer could bind to BCMA-expressing myeloma cells and can be used for targeted delivery of therapeutic RNA drugs. In addition, Fattal et al. selected a 2’-F-modified RNA aptamer that can bind to CD44 protein and CD44-expressing cells with high specificity and affinity [127]. Surprisingly, the conjugation of anti-CD44 aptamers to PEGylated liposomes improved their binding affinity [128]. Subsequently, scientists developed a siRNA delivery nanoplatform based on liposomes conjugated with anti-CD44 aptamers (Figure 8d) [129]. This nanoplatform is able to actively target CD44 which is highly expressed in triple-negative breast cancer cells for efficient gene silencing both in vitro and in vivo.

### 3.5. Locked Nucleic Acids (LNA)

#### 3.5.1. Molecular Structure of LNA

Locked nucleic acids, also known as bridged nucleic acids (BNA), were originally developed in the late 1990s [130,131]. LNA refers to modified nucleic acid with a methylene bridge between the 2′-oxygen and 4′-carbon of the ribose sugar that locks in an RNA-like, C3′-endo conformation. Thus, the conformational flexibility of the ribose is drastically limited. Nevertheless, LNA can still form duplexes with complementary DNA or RNA via Watson–Crick base-pairing rules. In addition, the binding affinity of LNA to DNA/RNA is quite high due to the entropic constraint imposed by the 2′-4′ linkage. In general, incorporation of each LNA nucleotide into the duplex increases the Tm by 3 to 9 °C, depending on the ON position and sequence compositions. The LNA/RNA duplex is a poor substrate of RNase H. Nevertheless, carefully designed antisense LNA with improved binding and target accessibility can elicit RNase H activity and mediate degradation of the mRNA [132,133,134]. Free LNAs can be taken up by cells via gymnosis and exhibit potent activity in nearly all cell types [133]. In addition, ONs with incorporated LNA monomers are commercially available in combination with other chemical modifications and labels. However, LNA-modified ONs occasionally lead to severe hepatotoxicity in animals, raising systematic toxicological concerns [15]. Accordingly, various LNA derivatives with unique binding and chemical features have been synthesized to resolve this issue [132].

#### 3.5.2. Synthesis of LNA

Synthesis of LNA monomers has been reported using a linear or convergent strategy [135]. For the linear approach, LNA-U and LNA-A monomers have been synthesized but their yields are relatively low. On the other hand, the convergent strategy has enabled synthesis of LNA monomers containing natural nucleobases with higher yield and superior scalability [136]. Dimethoxytritylation of 5′-oxygen and phosphitylation of 3′-oxygen leads to the synthesis of protected LNA phosphoramidite derivatives that are suitable for incorporation into ONs. The insertion of LNA phosphoramidites into ONs can easily be conducted via automated solid-phase synthesis with standard reagents and coupling protocols on a commercial DNA synthesizer. Due to the excellent compatibility of the automated chemical synthesis, LNA phosphoramidites can be incorporated with natural nucleotides or combined with other chemically modified nucleotides [136]. Therefore, LNA phosphoramidites and ONs with LNA modifications are now commercially available.

#### 3.5.3. Biomedical Applications of LNA

LNA has been widely investigated in gene regulation and therapy. For example, Wengel et al. demonstrated LNAs as antisense agents [137]. The LNA/DNA copolymers induced efficient antisense activity toward DOR mRNA in rat brain. In addition, Gait and coworkers showed that introducing LNA modification and other chemical modifications into ONs resulted in specific and efficient inhibition of Tat-dependent in vitro transcription in HeLa cells [138]. LNA has also been used to modify DNAzymes or siRNAs to achieve improved efficacy. Jadhav and coworkers showed that the LNA-modified 10–23 DNAzymes (termed antagomirzymes) had enhanced miRNA cleavage activity [139]. They were further evaluated for specific silencing of miRNA in vitro and in vivo. Mook et al. reported that minimal LNA modifications at the 3′ end of siRNA could effectively stabilize the siRNA [140]. However, multiple LNA modifications may result in decreased efficacy. Reduced off-target gene regulation was also found in vivo compared with naked siRNA.

**Figure 9:**
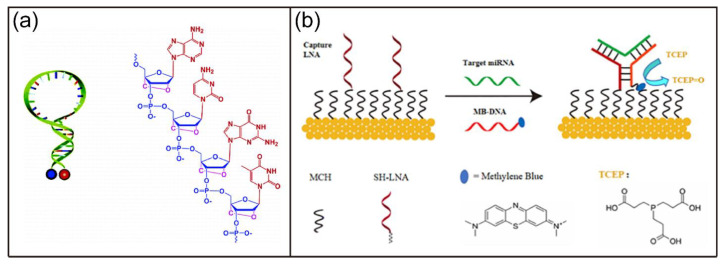
LNA-based biosensors. (**a**) LNA-based molecular beacons. Reprinted with permission from [141]. Copyright 2005, American Chemical Society. (**b**) A three-way junction DNA-based electrochemical biosensor for miRNA detection using LNA as the capture probe. Reprinted with permission from [143]. Copyright 2021, Elsevier.

In addition, a variety of LNA-based biosensors with high sensitivity and specificity have been developed. For example, Wang et al. developed a novel molecular beacon (MB) based on LNA bases (Figure 9a) [141]. The LNA MB had high thermal stability, superior single base mismatch discrimination capabilities, strong nuclease resistance, and did not bindwith single-stranded DNA binding proteins. Most recently, Feng and coworkers reported a three-way junction DNA-based electrochemical biosensor for miRNA detection using LNA as the capture probe (Figure 9b) [142]. The developed biosensor were highly sensitive and detected miR-2 with a detection limit of 77 aM. Moreover, nanoparticle-based LNA biosensors have also been explored. For example, Lin et al. reported a photothermal (PT) biosensor based on LNA, DNA walker, and AuNPs [143]. In their design, the target sequence activated the DNA walkers, leading to the formation of enzyme recognition site via the hybridization of walker strand and substrate strand. After substrate recognition, the ssDNA was released upon endonuclease cleavage and triggered the next round of cleavage, resulting in the improved stability of AuNPs against salt-induced aggregation. There was a linear relationship between temperature changes of the biosensor target concentrations due to the different PT effects of aggregated and dispersed AuNPs. The developed biosensor exhibited sensitive detection of p53 DNA sequence with a detection limit of 60 pM.

Aptamers bearing LNA-modified nucleotides have also been developed and investigated. For example, Darfeuille et al. introduced LNA modifications into RNA aptamers targeting HIV-1 TAR RNA [144,145]. The obtained LNA-modified aptamers had a similar binding affinity as the parent RNA aptamer but with strong nuclease resistance. Other studies have showed that the LNA modification influences the binding affinity of the original aptamers [146,147].

### 3.6. Threose Nucleic Acid (TNA)

#### 3.6.1. Molecular Structure of TNA

(3′,2′)-α-L-threose nucleic acid or threose nucleic acid (TNA), which was first synthesized and investigated by Eschenmoser and colleagues in 2000, has drawn remarkable interest in the past two decades [148]. In TNA, an unnatural four-carbon threose sugar substitutes the natural five-carbon ribose sugar in DNA while the nucleobases and phosphodiester bonds are unchanged. Thus, TNA has a five-atom backbone repeating unit connected by phosphodiester linkages occurring at the 2′ and 3′ positions of the threose ring, which are different from the phosphodiester linkages at the 3′ and 5′ positions found in DNA and RNA.

Despite a backbone that is one atom shorter, TNA can still form stable antiparallel duplex structures with complementary DNA, RNA, and itself that follows the Watson–Crick base-pairing rules. The binding affinity of TNA to RNA is stronger than to DNA [148,149]. Egli and coworkers investigated this phenomenon via X-ray analysis [150]. TNA residues in the DNA strand adopt the A-form rather than the B-form conformation. Thus, TNA hybridizes more strongly to RNA than DNA. In another study, Jaun et al. used solution-state nuclear magnetic resonance (NMR) spectroscopy studies to show that the TNA duplex adopts A-form helical geometry [151]. Their results also support the idea that TNA forms a more stable duplex with RNA than DNA. Later, Horn and coworkers fully explained why TNA binds more favorably with RNA than with DNA [152]. Using NMR they studied the structural and dynamic properties of TNA/RNA and TNA/DNA duplexes, isothermal titration calorimetry (ITC), ultraviolet (UV) spectroscopy, and circular dichroism (CD). They found that TNA facilitates the duplex structure, forcing an A-form helical geometry. NMR analysis of the kinetic and thermodynamic parameters for the individual base-pair opening events showed asymmetric fluctuations of the TNA/DNA duplex. Thus, DNA is not able to fully fit the conformational constraints of the rigid TNA backbone, leading to a less stable TNA/DNA duplex.

Other groups have also investigated the factors that affect the thermal stability of the TNA-based duplex. Heemstra and coworkers investigated 14 TNA/DNA duplexes and found that the purine content in TNA significantly influences the thermal stability of TNA/DNA duplexes [153]. In general, a lower TNA purine content results in a relatively lower Tm compared with DNA/DNA or RNA/DNA duplexes, while TNA/DNA duplexes with higher TNA purine content are more stable. This is because a higher TNA purine content leads to a TNA/DNA conformation similar to A-form helical geometry, while lower TNA purine content leads to a B-form conformation. The same group also showed that the thermal stability of DNA/TNA or DNA/RNA duplex can be affected by the change in DNA pyrimidine content [154]. The data also suggested that TNA behaves as a kinetic analogue of RNA. Zhang et al. showed that the presence of excess salt affects the thermodynamic stability of the base flipping in the TNA duplex, suggesting the necessity of adding sufficient salts in the simulation scheme [155]. Weber et al. showed that the GC base pairs in TNA/DNA duplexes have much weaker hydrogen bonds compared with RNA/DNA duplexes, while the AT pairs have nearly identical hydrogen bond strengths [156]. In addition, Eschenmoser and coworkers showed that replacement of adenine by 2,6-diaminopurine significantly enhances the thermal stability of TNA/TNA, TNA/RNA, and TNA/DNA duplexes [157]. They also showed that TNA analogues with chemical modification of the phosphodiester backbone can still possess the Watson–Crick base-pairing capability [158].

The construction of higher-order non-canonical structures based on TNA has also been established. Chaput and coworkers demonstrated the formation of a parallel stranded G-quadruplex structure that is fully composed of TNA [159]. Native polyacrylamide gel electrophoresis (PAGE), CD, and NMR analysis confirmed the formation of stable four-stranded helical structures. This TNA-based G-quadruplex has similar thermal stability to equivalent DNA G-quadruplexes.

#### 3.6.2. Synthesis of TNA

As mentioned, Eschenmoser et al. first synthesized TNA monomers and polymers [148]. They established a synthesis scheme for the preparation of TNA phosphoramidites, which was a crucial milestone for the future chemical solid-phase synthesis of TNA ONs using standard cyanophosphoramidite chemistry. This approach, however, has several disadvantages including low yield, numerous purification steps, and poor regioselectivity. Accordingly, Chaput et al. proposed an optimized synthesis scheme for L-threofuranosyl nucleosides [160]. Fewer purification steps and increased yield in this approach allow the synthesis of TNA monomers in gram-scale quantities. Researchers further developed synthesis strategies for robust and safe production of TNA nucleosides, phosphoramidites, triphosphates, and analogues, enabling the development of functional TNA molecules with expanded chemical diversity and enhanced physiochemical properties [161,162,163,164,165,166,167]. In addition, our group reported using 2-cyanoethyl N,N,N’,N’-tetraisopropylphosphoramidite for the cost-effective synthesis of TNA polymers [168].

Enzymatic synthesis of TNA has also been established. In an early study, Szostak et al. screened various DNA polymerases for activity on a TNA template and identified several polymerases with good ability to copy limited stretches of TNA [169]. Later, the Szostak group and the Herdewijn group reported several polymerases with the ability to synthesize TNA using a DNA template [170,171]. Although the reported polymerases could only synthesize DNA or TNA with limited length, they brought the idea of genetic information exchange between DNA and TNA to reality. Thereafter, Szostak et al. reported Therminator DNA polymerase, a variant of the 9°N DNA polymerase, for efficient and faithful DNA-directed TNA polymerization [172]. Kinetic analysis showed that Therminator DNA polymerase can recognize both a TNA primer and tNTP substrates [173]. Despite the changes in the geometry of the reactants, Therminator DNA polymerase is still an effective catalyst for TNA polymer synthesis. Therminator DNA polymerase, however, cannot transcribe DNA templates into TNA when the substrate is changed to a large pool of random sequences. To solve this problem, Chaput and coworkers proposed a primer extension assay to examine TNA synthesis under various conditions and established an L3 library as an efficient design strategy for generating pools of full-length TNA molecules [149]. They also reported a transcription and reverse transcription system using commercial enzymes for TNA replication with about 380-fold enrichment after one cycle of transcription, selection, reverse transcription, and amplification [8]. Recently, Chaput et al. reported engineered Kod-RI polymerases, which are the most efficient TNA polymerases developed to date for TNA synthesis and analysis of the structural basis [174,175]. In addition, the same group reported the systematic screening and optimized reaction condition of natural DNA and RNA ligases, and identified the bacteriophages, T7, T4, and T3 DNA ligase, as enzymes for synthesis of TNA-DNA, DNA-TNA, and TNA-TNA ONs via template-directed ligation [176].

#### 3.6.3. Biomedical Applications of TNA

Chaput et al. firstly studied the physiological stability of TNA [177]. They showed that TNA remains undigested after incubation in 50% human serum or human liver microsomes for 7 days and is highly resistant to a strong degradative snake venom phosphodiesterase (SVPDE). TNA can also protect internal DNA residues and shield complementary RNA strands from nuclease digestion. These results demonstrate the excellent physiological stability of TNA, which is essential for the development of biologically stable nucleic acid agents for biomedical applications.

**Figure 10:**
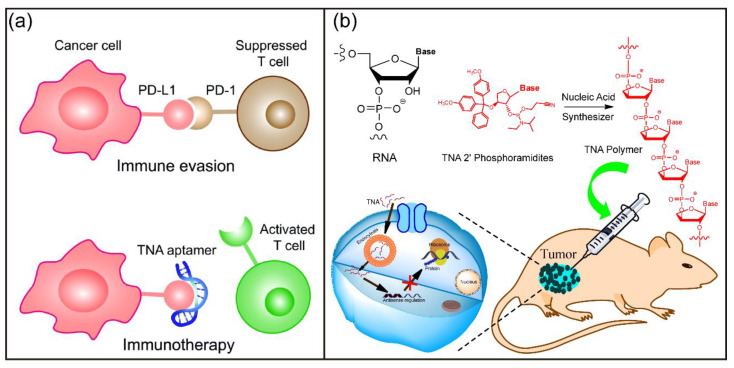
TNA for biomedical applications. (**a**) Schematic illumination of TNA aptamer targeting PD-L1 for cancer immunotherapy. Reprinted with permission from [183]. Copyright 2020, Royal Society of Chemistry. (**b**) Chemical structure of TNA polymers and the schematic illumination of TNA ONs for antisense cancer therapy. Reprinted with permission from [186]. Copyright 2019, American Chemical Society.

In 2012, Chaput and coworkers applied Darwinian evolution techniques for in vitro selection of TNA molecules with an arbitrary specific function, they generated the first TNA aptamer that targets human thrombin with high affinity and specificity [149]. The work demonstrated that TNA could fold into tertiary structures with retained chemical functions, suggesting TNA as an RNA progenitor in the pre-RNA world. After that, various TNA aptamers with the capability of binding to small molecules and large proteins have been selected [178,179,180,181,182,183]. These TNA aptamers have remarkable thermal and biological stability. In particular, Yu et al. demonstrated that the selected programmed death ligand 1 (PD-L1)-targeting TNA aptamer N5 effectively inhibited the programmed cell death protein 1 (PD-1)/PD-L1 interaction in vitro (Figure 10a) [181]. Furthermore, TNA aptamer N5 could specifically accumulate at the tumor site after systemic administration into a colon cancer xenograft mouse model and significantly suppressed tumor growth with negligible side effects. This work suggests that TNA-based agents can be developed into XNA immune checkpoint inhibitors for cancer immunotherapy.

TNA polymers for gene regulation and therapy have also been investigated. Our group showed that biostable and biocompatible TNA polymers could penetrate and accumulate in various living cell lines without transfection [168]. In addition, the designed antisense TNA-based polymers significantly suppressed green fluorescent protein (GFP) gene expression, suggesting TNA as an alternative to traditional ASOs. Subsequently, we demonstrated the application of TNA polymers for inhibition of target gene expression in vitro and antisense cancer therapy in vivo (Figure 10b) [184]. Chaput et al. evaluated TNA in nucleic acid therapeutics and found that a cytosine-phosphate-guanine (CpG) ON sequence composed entirely of TNA activated innate immune responses with a slight induction of relevant mRNA signals and robust B-cell activation [185]. In addition, Ding and coworkers reported a PCR-based strategy for the construction of a terminal-closed linear gene with a TNA loop modified primer pair [186]. The developed linear gene had enhanced enzymatic resistance and serum stability and potently and persistently expressed enhanced green fluorescent protein (EGFP) gene in eukaryotic cells. These studies provide novel techniques for the development of gene therapy.

Recently, TNA-based catalysts (or enzymes) have been reported. For example, Chaput and coworkers demonstrated that the introduction of TNA and 2′-fluoroarabino (FANA) modifications into classic DNAzyme 10–23 (X10-23) significantly enhanced biological stability and catalytic activity [187]. The developed X10-23 efficiently and persistently silenced gene activity via degradation of mRNA molecules in cultured mammalian cells. They also demonstrated the use of X10-23 to knock down allele-specific mRNA sequences in disease cells and to detect the viral pathogen responsible for COVID-19 with a detection limit of ≤20 aM [188,189]. To attain clear comprehension, a summary of the discussed nucleic-acid-based biosensors in this review is shown in Table 2.

Moreover, Yu et al. identified the first TNA enzyme with RNA ligase activity via in vitro selection [190]. The isolated TNA enzyme T8-6 could catalyze the formation of a 2′-5′ phosphoester bond between a 2′,3′-diol and a 5′-triphosphate group with UA|GA residues at the ligation junction. It could tolerate variations at other substrate positions. They further demonstrated successful formation of functional RNA molecules with a site-specific 2′-5′ linkage such as a hammerhead ribozyme via a T8-6-catalyzed ligation. This work provides experimental support for TNA as a potential pre-RNA genetic polymer and offers an alternative molecular tool for biological and biomedical research.

## 4. Nucleic Acid Nanotechnology

The improved understanding of nucleic acid structure has not only illuminated the fundamental basis of nucleic acid biological functions, but has also provided a novel concept of self-assembly technology and is versatile material for designing nanometer-scale structures. In general, by designing the sequence of nucleic acid strands, predictable and programmable self-assembled two-dimensional (2D) or even 3D nanostructures can be obtained [18,191]. DNA tiles and DNA origami are two fundamental elements in DNA self-assembly nanotechnology [17]. DNA tiles are relatively simple. They are usually composed of only a few DNA ONs. DNA origami, however, sequesters hundreds of short DNA strands that recognize and bind to a long viral ssDNA as a scaffold to form the predesigned nanostructure [17]. Today, many 2D or 3D DNA origami nanostructures have been designed and constructed where play a vital role in DNA nanotechnology [12]. In addition, RNA molecules can be designed to fold or assemble into nanostructures just like DNA. Nevertheless, the research in the RNA nanotechnology field has focused on designing higher-order RNA structures through natural RNA motifs and association methods [2,192].

**Figure 11:**
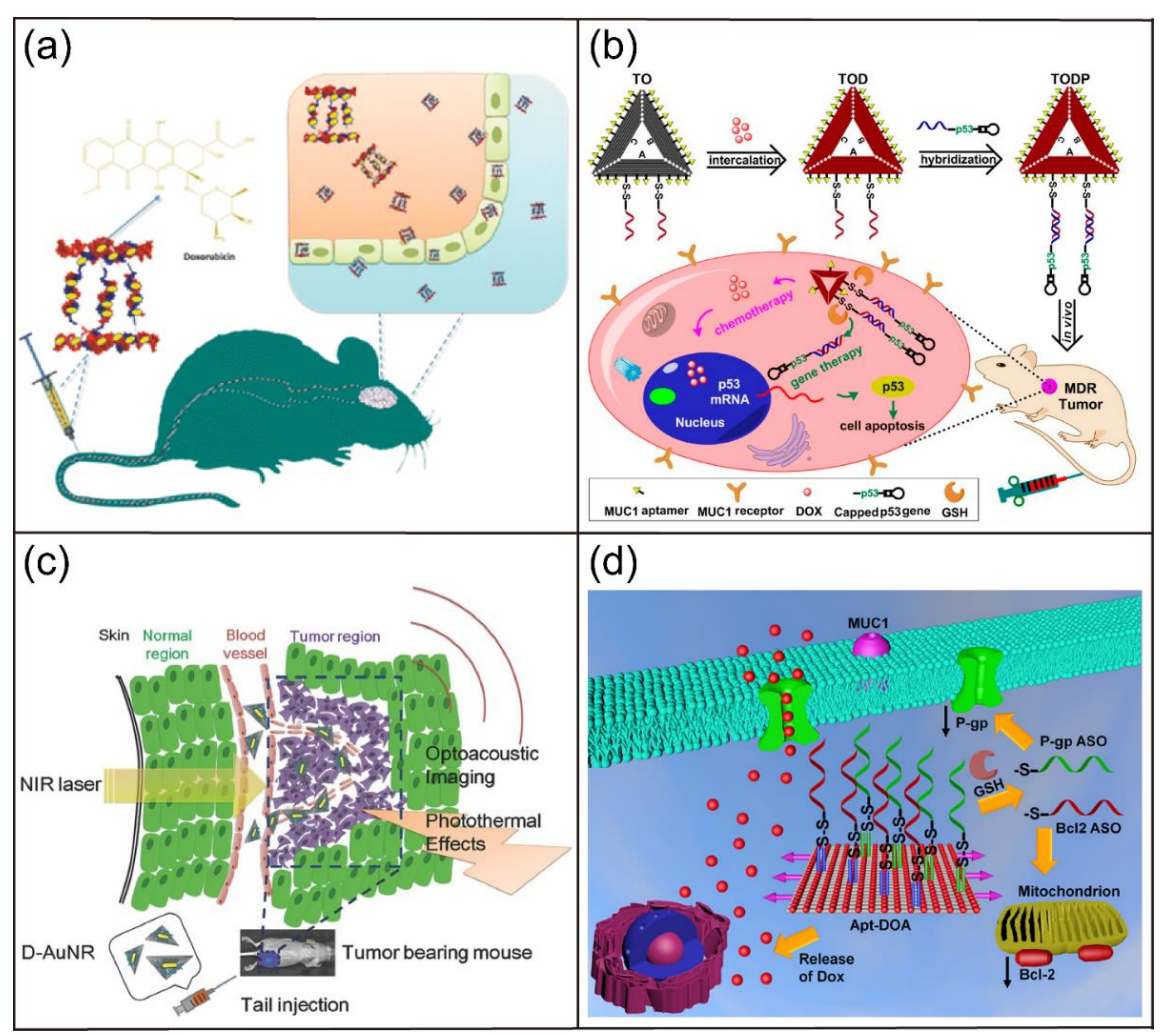
Nucleic-acid-based nanoplatforms for drug delivery. (**a**) Self-assembled DNA nanocage for BBB penetration and drug delivery in brain cancer therapy. Reprinted with permission from [196]. Copyright 2020, American Chemical Society. (**b**) A DNA nanostructure-based platform for simultaneous delivery of linear p53 gene and anticancer DOX for combined therapy. Reprinted with permission from [197]. Copyright 2018, American Chemical Society. (**c**) DNA origami loaded with gold nanorods for optoacoustic imaging and photothermal therapy. Reprinted with permission from [198]. Copyright 2016, Wiley-VCH. (**d**) Aptamer-functionalized DNA origami for codelivery of DOX and two ASOs for enhanced therapy. Reprinted with permission from [199]. Copyright 2020, American Chemical Society.

Owing to their intrinsic biological and chemical properties, self-assembled nucleic acid nanostructures have been used as delivery platforms for various therapeutic drugs, including chemotherapeutic drugs, biomacromolecules, and nanoparticles [12,18]. For example, our group developed a lipid-functionalized DNA nanocage (LNC)-based nanoplatform for mitochondrial delivery of drugs [193]. The LNCs had enhanced cellular uptake via temperature-, energy-, and clathrin-dependent endocytosis. The developed nanoplatform enabled the delivery of doxorubicin (DOX) to the mitochondria and induced significant cytotoxicity to MCF-7 cells. As shown in Figure 11a, our group also developed self-assembled DNA nanocages functionalized with BBB-targeting ligands for drug delivery in brain cancer therapy [194]. The DNA nanocages could deliver the chemotherapeutic drug, DOX, across the BBB and significantly inhibit U87 MG tumor growth in vivo. Nucleic-acid-based platforms have also been show to be promoting for the delivery of large cargoes such as biomacromolecules and nanoparticles. For example, Ding et al. introduced a facile technique to construct a delivery system based on a DNA nanostructure containing a linear p53 gene and chemotherapeutic drug, DOX, (Figure 11b) [195]. In vitro and in vivo studies show that self-assembled DNA nanostructures are efficient gene delivery system in multidrug-resistant MCF-7R cancer cells resulting in remarkable inhibition of tumor growth without apparent systemic toxicity. As shown in Figure 11c, Du and coworkers designed a theranostic platform based on DNA origami nanostructures, which were loaded with gold nanorods for enhanced in vivo optoacoustic imaging and photothermal therapy [196]. Most recently, Pan et al. reported aptamer-functionalized DNA origami for targeted codelivery of the chemotherapeutic drug, DOX, and two ASOs into drug-resistant cancer cells (Figure 11d) [197]. The developed nanoplatform circumvented the multidrug resistance in HeLa/ADR and MCF-7/ADR cells and significantly enhanced therapeutic effects.

Recently, stimuli-responsive nucleic-acid-based nanomaterials have been widely designed and studied [198]. In general, external molecular stimuli inputs, such as small molecules, enzymes, protons, metal ions, heat, nucleic acids, and light, are added to the DNA-based nanomaterials to trigger the reconfiguration of the DNA systems and achieve certain functions [199,200]. G-rich strands can be stabilized into G-quadruplexes in the presence of metal ions. For example, our group reported a strategy to extend and contract DNA nanocages based on G-rich strands [201]. The contraction and extension of these developed DNA nanocages can be regulated via the reversible formation and deformation of G-quadruplex in the presence of K^+^ ions and chelating agents. By integrating three human telomeric strands, the developed DNA nanocages can function as horseradish peroxidase, mimicking DNAzymes for colorimetric detection of cholesterol with high sensitivity and enzymatic stability. Subsequently, our group reported a novel strategy for metal ion-responsive self-assembly of polymeric DNA nanostructures via the introduction of G-quartet toeholds within the edges of the discrete DNA building blocks as adhesive units [202]. The morphology of these DNA nanostructures could be reversibly manipulated via parallel or antiparallel formation of G-quadruplexes. Furthermore, this strategy could regulate the cycling of DNA nanostructures between discrete and polymeric states upon the sequential introduction of cations and chelating agents. Nanoscale DNA assemblies that are responsive to other stimuli have also been reported. For example, Gu et al. reported a pH-responsive DNA nanocarrier for the targeted delivery of anticancer drug, DOX, into tumor cells [203]. This DNA-based nanocarrier consisted of deoxyribonuclease-degradable DNA nanoclew and an acid-responsive nanocapsule. DOX was loaded in the nanoclew via intercalation and DNase I was encapsulated in the nanocapsule. The positively charged nanocapsule could then be embedded into the nanoclew via electrostatic interactions. When the DNA nanocarriers were internalized by cancer cells and reached the acidic endosomes, the nanocapsule was degraded releasing the DNase I, leading to the degradation of the nanoclew and subsequent release of anticancer DOX. The overall outcome was enhanced therapeutic efficacy. In another study, Han and coworkers constructed a DNA nanorobot for responsive autonomous anticoagulation in human plasma [204]. Within this nanorobot, a barrel-shaped DNA nanostructure was designed as a framework to protect the functional components. Three modular DNA building blocks were embedded inside as the computing core for sensing, thresholding, and regulation. The developed DNA nanorobot could respond to the thrombin and trigger autonomous anticoagulation with excess thrombin. Furthermore, our group also reported a DNA-based OR logic gate (D-OR) that could be operated by one- or two-photon irradiation. Our DNA logic gate could be easily reset to the original state and operated repeatedly. This work has contributed to photoresponsive DNA-based systems in computing, optical communication, and biology [205]. In addition, our group also developed UV and vis irradiation responsive DNA nanotube systems [206]. In this study, we achieved reversible conformational changes and switchable patterning of gold nanoparticles of DNA systems in response to UV or Vis irradiation.

## 5. Conclusions and Perspectives

The development of nucleic acid research offers an opportunity for the use of nucleic-acid-based agents for various biomedical applications. For instance, natural nucleic acids including ASOs, siRNAs, miRNAs, and aptamers are promising gene regulation tools and therapeutic agents for highly selective treatment strategies for human diseases [6]. In particular, aptamers have been used in a large number of sensing platforms as smart molecular probes for specific recognition of molecular targets including ions, small molecules, proteins, and cells [13]. While significant scientific advances in the development of nucleic-acid-based biomaterials as potential biomedical applications have been achieved, several important challenges remain.

DNA or RNA molecules including ASOs and siRNAs have emerged as a new generation of therapeutics, in the post-genome era, that can selectively shut down expression of proteins associated with diseases. So far, an antisense strategy has been widely used for potential cancer treatment because of its ability as a self-repairing system with reduce off-target toxicity. Downregulation of some therapeutic target genes including BCL-2, PTEN, PDCD4, and AKT [207,208,209] using the appropriate sequences of miRNAs or siRNAs together with molecular drugs could effectively inhibit the growth of certain cancer cells in culture and tumor xenografts [207,208,209,210,211,212,213]. Thus, gene silencing based on the RNAi technique has antisense power for cancer therapy [168,184,186,187]. However, the number of nucleic-acid-based antisense drugs approved by the U.S. FDA is still low, resulting in slow progress in the development of nucleic-acid-based therapeutics. Indeed, as drugs, they still suffer from several drawbacks such as instability, poor cellular uptake, and laborious syntheses. To improve the nuclease resistance, duplex-forming ability, and pharmacokinetic properties of RNAi-based therapeutics, the research community has put great efforts into the generating a series of nucleic acid analogues by chemically modifying the phosphodiester backbone, sugar moiety, or nucleobases of natural nucleic acids [15]. Chaput et al. reported that TNA can be used to protect the internal DNA residues and further shield complementary RNA strands from nuclease digestion [177]. Their finding shows the excellent physiological stability of TNA, which is significant for the development of highly stable nucleic acid agents for biomedical applications. Lipofectamine transfection is required for the delivery of these therapeutic antisense materials. This non-viral treatment is highly restricted in the treatment of some diseases such as cancers. At present, some of the modified nucleic acid analogues such as TNAs are substantially uptaken in a number of cancer cell lines without the need of additional transfection treatment to induce gene silencing and antitumor effects in vivo [168,184]. Compared to conventional cancer therapy such as radiotherapy, surgery, and chemotherapy, we condifently envision that modified nucleic-acid-based antisense therapy will shortly start to play an important role in clinical practice.

Alternatively, an effective delivery strategy involved the use of nanocarriers against cancers is urgently needed in the clinical sector such as cancer centers and hospitals. Natural or synthetic compounds including liposomes, polymers, cationic lipids, and lipid-polymer hybrids have been proposed as nanocarriers for systemic delivery of siRNA/miRNAs into the cells [208,212,213,214,215,216]. Unfortunately, drug loading efficiency of polymeric nanoparticles is not sufficiently high while the loaded therapeutic drugs had either very low or very fast release kinetics after administration in the blood stream [217,218]. Developing stimuli-responsive nanomaterials as versatile and multifunctional nanocarriers for remote-controlled release of therapeutic nucleic acid agents to desired sites will be highly noteworthy and innovative. Our group has successfully developed photoresponsive DNA nanocarriers that are able to change the morphology of the containers for potential drug release in response to light irradiation in a remote-controlled manner [206,219]. Recently, Yang‘s group reported the use of controllable association/dissociation of DNA/polyphenol nanoassemblies for cascade-responsive and sequential drug release in cancer cells [220]. Addition of tannic acid (TA)acts as a linker to mediate self-assembly of branched DNA tiles loaded with antisense DNA and DNAzyme to form a nanocomplex. Inside the acidic lysosomal environment, the nanoassembly is dissociated to release the TA and branched DNA. Subsequently, the DNase I and glutathione in cytosol trigger the release of antisense DNA and DNAzyme to inhibit cancer cell proliferation and cell migration, respectively. We believe that this work demonstrates a controllable association/dissociation method to address the conflict between sufficient drug loading and efficient drug release in living cells for disease therapy.

Additionally, the detection sensitivity of the aptamers can be greatly improved by conjugating aptamers with other nanomaterials. Work conducted by Zhang et al. introduced a binding-induced DNA assembly idea for the detection of targets with low concentrations in human blood samples [221]. We strongly believe that this detection strategy could be utilized for a large number of biomolecules including oligosaccharides, lipids, nucleic acids, pathogens, and viruses. Subsequently, Chen et al. applied a signal amplification strategy to develop an electrochemical aptasensor for improving the sensitivity of lysozyme detection [222]. In his design, a large number of ssDNA was loaded onto the AuNPs where they further link together to form a star-shaped trithiol system after adding thiocyanuric acid. This trithiol-functionalized Au system was hybridized to the anti-lysozyme aptamer which was immobilized onto the Au electrode, followed by [Ru(NH_3_)_6_]^3+^ binding to DNAs. The detection signal is indicated by a decrease in charge of the surface-bound [Ru(NH_3_)_6_]^3+^. Furthermore, immobilizing more than one aptamer in a single detection platform for simultaneous detection or diagnosis of more than one target/disease is highly recommended. As aptamers are short strands of oligonucleotides, we took advantage of this unique property to decorate them as part of the building blocks of programmable DNA-based assemblies for specific recognition of multiplex targets. We designed a single DNA nanotube with aptamer sequences that simultaneously recognized and discriminated three targets, thrombin, ATP, and insulin. [66]. This design approach eliminates the efforts of additional chemical functionalization or conjugation on the supports or substrates. We believe that DNA nanotechnology-based detection systems will be useful for cost-effective and fast detection of multiplexes in the near future.

Nowadays, large-scale production of nucleic acids/modified nucleic acid analogues is highly limited. They are usually chemically synthesized in the laboratory using DNA synthesizer, and the yield is in the nanomole or micromole ranges due to technical problems. To scale up the synthesis, we suggest creating a DNA-mimetic polymer by using a combination of molecular recognition, self-assembly, and synthetic polymer chemistry. This can result in an entirely novel class of “DNA-mimetic” polymers that, have the specificity and monodispersity of the parent DNA molecule, yet possess the desirable properties of synthetic polymers such as stability, can be readily synthesized in large quantities, facile functionalization, and improved solubility, and cellular uptake properties. To achieve this goal, we conducted a nucleobase-templated study to copy the chain length and polydispersity of living polymers to daughter conjugated polymers [223]. We created a thymine-containing polymer that exhibits narrow molecular weight distribution by living ring-opening metathesis polymerization. We then aligned the designed monomers, each with a complementary nucleobase along the parent template by hydrogen bonding interactions. Subsequently, templated Sonogashira polymerization was carried out to generate a daughter conjugated polymer. Importantly, we found that this daughter polymer strand had narrow molecular weight distribution. This study demonstrated that templated polymerization using nucleobase recognition is feasible to copy the polydispersity and molecular weight distribution from a parent template to a daughter synthetic strand. This is an important step to explore the concept of transferring nucleobase sequence from template to daughter strands in order to eventually mimic DNA information to fully synthetic polymers via typical polymerization reactions. In principle, this fully synthetic polymer will become cost-effective therapeutics because they can be easily and cheaply synthesized in commercially on an industrial scale, potentially in the tens of kilogram range.

RNAi-based therapeutics could definitely have great impacts and become viable in clinical applications if their systemic delivery is not limited to liver or kidney tissues. We suggest that the interactions of nucleic-acid-based drugs with living organisms should be widely investigated to address some major challenges in terms of elimination of both renal and reticuloendothelial clearance, improvement in extravasation and tissue perfusion, enhancement in cellular uptake and further endosomal uptake. For example, a major problem for gene therapy is poor tissue penetration and distribution of nucleic-acid-based drugs to generate a therapeutic effect despite the loaded drugs reaching the target cells/tumors. It is important to assess the penetration capability of chemically modified nucleic acid analogues into tumor tissues and their biodistribution properties.

## Figures and Tables

**Table 1:** Characteristics of different types of nucleic acid analogues.

Type	Duplex Formation	Nuclease Stability	RNase H Recruitment	Commercial Availability	Limitations
PS ONs	yes	increased	capable	yes	relatively unstable duplex
PNA	enhanced binding affinity	increased	incapable	yes	low aqueous solubility, self-aggregation
2′-OMe RNA	stronger binding affinity to RNA than DNA	increased	incapable	yes	reduced silencing activity of modified siRNA
2′-F RNA	enhanced binding affinity to RNA	not significantly increased	incapable	yes	other modifications required to enhance nuclease stability
LNA	increased	increased	poor substrate	yes	severe hepatotoxicity
TNA	stronger binding affinity to RNA than DNA	increased	incapable	no	Limited length in chemical synthesis

**Table 2:** A summary of nucleic-acid-based sensors.

Sensor	Target	Detection Limit/Range	Ref.
DNA-AuNPs nano-flares	Surviving mRNA	in vitro imaging	[51]
Graphene-DNAzyme	Cu^2+^	0.365 nM	[54]
Ribozyme-based biosensor	TPP	a few nM	[55]
DNAzyme sensor	Li^+^	in vitro imaging	[56]
electrochemical aptasensor	ATP	10 nM to 1 mM	[64]
Aptamer-modified graphene transistor	*E. coli*	10^2^ CFU/mL	[65]
Aptamer-modified DNA nanotube	thrombin, ATP, and insulin	~17.6 nM, ~116 nM, and ~55 nM	[66]
Aptamer-modified Ag_2_S nanodots	CTCs	6 tumor cells/mL	[68]
PNA electrochemical biosensor	DNA	10 pmol	[96]
PNA-graphene oxide	dsDNA	260 pM	[97]
PNA-AuNPs	single nucleotide polymorphism	2.3 nM	[98]
PNA-graphene oxide	miRNAs	~1 pM	[99]
LNA MB	single nucleotide polymorphism	NA	[141]
LNA electrochemical biosensor	miRNA	77 aM	[142]
LNA-modified PT biosensor	P53 DNA sequence	60 pM	[143]
TNA-based biosensor	SARS-CoV-2 RNA	≤20 aM	[189]

## Data Availability

No new data were created or analyzed in this study.
